# Study of *p53* gene alteration as a biomarker to evaluate the malignant risk of Lugol-unstained lesion with non-dysplasia in the oesophagus

**DOI:** 10.1038/sj.bjc.6603582

**Published:** 2007-02-06

**Authors:** K Kaneko, A Katagiri, K Konishi, T Kurahashi, H Ito, Y Kumekawa, T Yamamoto, T Muramoto, Y Kubota, H Nozawa, R Makino, M Kushima, M Imawari

**Affiliations:** 1 Second Department of Internal Medicine, Showa University School of Medicine, Tokyo, Japan; 2 Clinical Research Laboratory, Showa University School of Medicine, Tokyo, Japan; 3 Department of Pathology, Showa University School of Medicine, Tokyo, Japan

**Keywords:** *p53* mutation, Lugol staining, oesophagitis, dysplasia, endoscopy, precursor

## Abstract

Mutations of the *p53* gene are detected frequently in oesophageal dysplasia and cancer. It is unclear whether Lugol-unstained lesions (LULs) with non-dysplastic epithelium (NDE) are precursors of oesophageal squamous cell carcinoma (ESCC). To study the genetic alterations of NDE in the multistep process of oesophageal carcinogenesis, we determined the relationship between *p53* mutations and LULs-NDE. Videoendoscopy with Lugol staining was performed prospectively in 542 oesophageal cancer-free subjects. Lugol-unstained lesions were detected in 103 subjects (19%). A total of 255 samples, including 152 LULs (NDE, 137; dysplasia, 15) and 103 paired samples of normal staining epithelium, were obtained from 103 subjects. After extraction of DNA and polymerase chain reaction analysis, direct sequencing method was applied to detect mutations of the *p53* gene. The *p53* mutation was detected in five of 137 samples with LULs-NDE (4%) and in five of 15 samples with dysplasia (33%). A hotspot mutation was found in 20% of LULs-NDE with *p53* mutation and in 40% of dysplasia with *p53* mutation. In contrast, no *p53* mutations were found in 103 paired NDE samples with normal Lugol staining. In biopsy samples from oesophageal cancer-free individuals, the *p53* missense mutations containing a hotspot mutation were found in NDE, which was identified as an LUL. These findings suggest that some LULs-NDE may represent the earliest state of oesophageal squamous cell carcinoma in Japanese individuals.

Oesophageal squamous cell carcinoma (ESCC) is one of the most common carcinoma worldwide, with marked variation in its incidence rate among different countries, distinct geographic areas, and different ethnic groups (Parkin *et al*, 1988). Among oesophageal cancers in Japanese patients, 95% are squamous cell carcinomas (Registration Committee for Esophageal Cancer). In Western countries and Japan, heavy cigarette smoking and alcohol intake are the risk factors, whereas in the developing countries, exposure to dietary carcinogens and nutritional deficiencies are believed to be the major aetiologic factors (Yang, 1980; Von Rensburg, 1981; Parkin *et al*, 1988; Yokoyama *et al*, 1995). However, results from previous studies suggest that malignant transformation of human oesophageal epithelium is a multistage progressive process (Yang, 1980; Yang and Qiu, 1987; Qiu and Yang, 1988; Wang *et al*, 1990; Bennett *et al*, 1992; Wang *et al*, 1993; Greenblatt *et al*, 1994).

Characterisation of human oesophageal precancerous lesions at the molecular level is of critical importance to our understanding of the aetiology of ESCC and to the identification of useful biomarkers for prevention studies of that disease. Mutation analyses among high-risk Chinese populations have demonstrated that *p53* gene mutations occur at an early stage of oesophageal carcinogenesis, both in the setting of basal cell hyperplasia (BCH) and in dysplastic lesions (Bennett *et al*, 1992; Wang *et al*, 1993; Gao *et al*, 1994; Jaskiewicz and De Groot, 1994; Parenti *et al*, 1995). An early indicator of abnormality in individuals predisposed to ESCC is an increased proliferation of the oesophageal epithelial cells, morphologically manifested as BCH, dysplasia, and cancer *in situ*. Most of these lesions could be considered as precancerous lesions because of the presence of *p53* mutations (Yang, 1980; Yang and Qiu, 1987; Qiu and Yang, 1988; Wang *et al*, 1990; Mandard *et al*, 2000). But it is under debate whether BCH is a precancerous lesion for ESCC or not, as no hotspot mutations of the *p53* gene were found in BCH samples (Shi *et al*, 1999).

Although endoscopic detection for early ESCC is extremely important because of excellent 5-year survival rate (Yoshinaka *et al*, 1991; Kumagai *et al*, 1993), two-thirds of oesophageal intraepithelial carcinomas have been overlooked by conventional endoscopy alone (Sugimachi *et al*, 1989). A simple technique of spraying Lugol solution in the oesophagus is highly sensitive for identifying dysplasia and intraepithelial carcinoma (Mori *et al*, 1993; Meyer *et al*, 1997; Dawsey *et al*, 1998). According to the Lugol staining pattern, completely ‘unstained’ areas were found in approximately 90% of high-grade dysplasia and carcinoma, whereas approximately 90% of staining areas, which were less intensely stained than normally stained epithelium, were non-dysplastic lesions and the remaining 10% were dysplasia (Mori *et al*, 1993). Therefore, Lugol-unstained lesions (LULs) are detectable not only in dysplasias and carcinomas but also in non-dysplastic areas, for example with oesophagitis, or in the setting of Barrett's oesophagus (Sugimachi *et al*, 1989; Dawsey *et al*, 1998). In contrast, Lugol staining methods were not used in most studies regarding *p53* mutational status in oesophageal precancerous lesions such as dysplasia, BCH, and esophagitis (Yang and Qiu, 1987; Qiu and Yang, 1988; Wang *et al*, 1990; Bennett *et al*, 1992; Wang *et al*, 1993; Gao *et al*, 1994; Greenblatt *et al*, 1994; Jaskiewicz and De Groot, 1994; Parenti *et al*, 1995; Shi *et al*, 1999; Mandard *et al*, 2000).

Resected specimens from cancer patients were used in the analysis of the *p53* mutations in the previous studies (Yang, 1980; Yang and Qiu, 1987; Qiu and Yang, 1988; Wang *et al*, 1990; Shi *et al*, 1999; Mandard *et al*, 2000). Little information is available regarding the *p53* mutational status in the Lugol-unstained lesions with non-dysplastic epithelium (LULs-NDE) of oesophageal cancer-free subjects. To determine the genetic alterations in the early stage of oesophageal carcinogenesis, oesophageal cancer-free subjects should be selected. Endoscopic detection of oesophageal precancerous lesions and molecular diagnosis is of clinical importance to identify high-risk patients and to prevent the development of ESCC. We carried out a prospective study of the *p53* mutational status of both LULs-NDE and paired samples of normal Lugol staining areas from endoscopic biopsy samples obtained after spraying the oesophagus with Lugol solution.

## MATERIALS AND METHODS

### Study design

To investigate whether LULs-NDE were related to the carcinogenesis of oesophageal squamous epithelium or not, the *p53* mutational status in LULs-NDE was analysed prospectively. Secondary end points were to elucidate whether BCH is related to oesophageal carcinogenesis through the *p53* mutational status and examine malignant potential in multiple LULs. Recruited subjects were composed of oesophageal cancer-free individuals who visited our hospital for a health checkup between April 1999 and March 2001. Subjects were recruited on the basis of the following criteria: male and female individuals, age in the range of 20–80 years, the subjects performance status being ‘zero’ according to Eastern Cooperative Oncology Group (ECOG), and the subjects with no symptoms of dysphagia, abdominal pain, chest and/or back pain, or vomiting were eligible. As LULs can be caused by reflux oesophagitis, subjects with heartburn and those receiving proton pump inhibitor therapy were excluded. Subjects who had active malignant disease, and who had undergone oesophagectomy or chemoradiotherapy for ESCC, were excluded. After endoscopic observation, subjects who had oesophageal varices, Barrett's oesophagus, or reflux oesophagitis were also excluded. Although heavy cigarette smoking and alcohol intake are the major risk factors of ESCC, whether the oesophageal precancerous lesions are caused by such daily consumption or not is uncertain. Therefore, the subjects were not selected based on risk factors such as smoking and alcohol drinking. Participants were interviewed using structured questionnaires, which included queries about smoking and drinking status after recruitment. All subjects gave informed consent for participation in the study. The study protocol was approved by the Human Ethics Review Committee of Showa University School of Medicine.

### Patient population

A total of 599 subjects were recruited: 542 subjects matched the recruitment criteria and 42 subjects were excluded from the study. The reasons for exclusion were symptom-free reflux oesophagitis in 15 subjects, Barrett's oesophagus in five subjects, gastric carcinoma in one subject, and rejection to the study in 21 subjects. The mean age was 61 years, ranging from 20 to 80 years, and the male to female ratio was 274/268. Of the 542 subjects, 157 (29%) and 130 (24%) had daily consumption of cigarette and alcohol, respectively.

### Endoscopic examination

**Figure 1:**
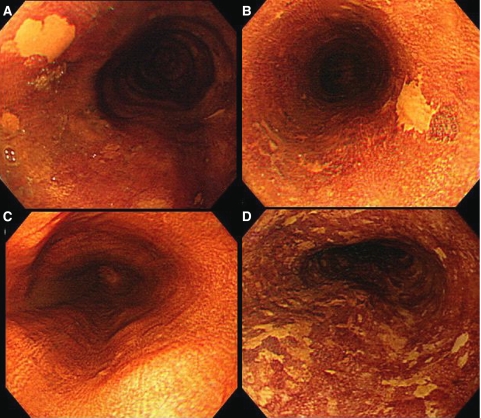
(**A**) Endoscopic findings of a Lugol-unstained lesion. This lesion was completely unstained. The lesion was oval and 4 mm in diameter. (**B**) Endoscopic findings of a Lugol-unstained lesion. This lesion was completely unstained. The lesion was irregular in shape and 6 mm in diameter. (**C**) Endoscopic findings of normal Lugol staining epithelium without a Lugol-unstained lesion. (**D**) Endoscopic findings of multiple Lugol-unstained lesions. Many irregular lesions that were stained less intensely than normal Lugol staining epithelium were located in one endoscopic view.

Videoendoscopy (Q240, Olympus, Tokyo, Japan) following Lugol solution spraying was performed on all oesophageal cancer-free subjects who matched the recruitment criteria. After ordinary endoscopic observation, 5–10 ml of 2.0% glycerin-free Lugol's iodine solution, which was a brown liquid consisting of 2.0 g potassium iodine and 4.0 g iodine in 100 ml distilled water, was sprayed from the gastroesophageal junction to the upper oesophagus using a plastic spray catheter (washing tube PW-5L; Olympus, Tokyo, Japan) passed through the biopsy channel of the endoscope. The whole oesophagus was observed again and epithelial areas were categorised as unstained, normally stained, or overstained. We defined LULs as those areas either staining less intensely than normally stained epithelium or completely unstained (Figure 1A–C); this group of lesions included carcinoma, dysplasia, and oesophagitis. When 10 and more than 10 LULs were detected in one endoscopic view, we defined them as multiple LULs (Figure 1D). Biopsies were taken under endoscopic guidance for LULs and paired normal Lugol staining background epithelium. The background epithelium specimens were obtained 1–5 cm away from LULs. We confirmed that samples were correctly taken from LULs during endoscopic observation. Histologic diagnosis among normal epithelium, oesophagitis, BCH, dysplasia, and carcinoma was made according to previously described definitions (Dawsey *et al*, 1994). Histologic features were evaluated by a pathologist in our hospital.

### DNA extraction

Ten 2-*μ*m-thick sections were obtained from each archival block of formalin-fixed and paraffin-embedded dysplastic and non-dysplastic tissue. One section of each block was stained with haematoxylin and eosin. The percentage of neoplastic cells was estimated by light microscopic evaluation, and the samples containing a minimum of 60% dysplastic cells were chosen. DNA samples were extracted by the ethanol/xylene method from the remaining nine sections (Goelz *et al*, 1985).

### Analysis of the *p53* gene

**Figure 2:**
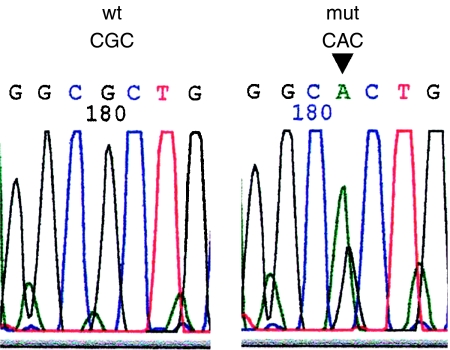
Mutation of the *p53* gene at codon 175 in exon 5 was shown in electropherograms. Base changed from CGC to CAC (black arrow). wt, wild type; mut, mutation.

Specimens were mixed with 50 *μ*l of digestion buffer (0.04% proteinase K, 10 mM Tris-HCl at pH 8.0, 1 mM EDTA, and 1% Tween 20) and incubated at 37°C for 18 h. The DNA fragments were analysed for mutations in *p53* exons 5, 6, 7, and 8, as described in our previous report (Makino *et al*, 2000). Primers used for polymerase chain reaction (PCR) amplification of the *p53* gene were as follows: for exon 5, 5′-TTCACTTGTGCCCTGATTTC-3′ and 5′-CTCTCCAGCCCCAGCTGCTC-3′; for exon 6, 5′-ATTCCTCACTGATTGCTCC-3′ and 5′-TCCTCCCAGAGACCCCAGTT-3′; for exon 7, 5′-ACAGGTCCTCCCCAAGGCGCA–3′ and 5′-TGTGCAGGGTGGCAAGTGGCT-3′; for exon 8, 5′-GTAGGACCTGATTTCCTTACTGCC-3′ and 5′-CTTGGTCTCCTCCACCGCTTCTTG-3′. Polymerase chain reaction conditions were set as described in our report (Makino *et al*, 2000). The PCR products were purified and directly sequenced using a 3100 sequencing machine (Applied Biosystems, Foster City, CA, USA). Peak patterns were analysed using Sequencing Analysis Software (Applied Biosystems, Foster City, CA, USA), and mutations and amino-acid changes were identified (Figure 2). To ensure reproducibility of our data, direct sequencing was performed at least twice in DNA samples.

### Statistical analysis

As LULs were found in approximately 20% of 1000 patients undergoing routine endoscopy in our previous experience, sample size was estimated to be 500 patients to collect at least 100 patients with LULs. To avoid bias, the data regarding the detection of *p53* mutation in LULs and their paired normal Lugol staining areas were re-identified for genetic and clinicopathologic analyses. These data were then matched after the genetic and clinicopathologic analyses were completed. The significance of differences between the two groups was assessed by the *χ*^2^ test or Wilcoxon rank-sum test. *P*-value of less than 0.05 was considered significant.

## RESULTS

### Characteristics of subjects

Out of 542 subjects, LULs were found in 103 (19%).The mean age was 62 years, ranging from 25 to 80 years, and the male to female ratio was 63/50. Of the 103 subjects, 35 (34%) and 31 (30%) had a daily habit of cigarette smoking and alcohol drinking, respectively. No significant difference in the frequency of daily cigarette smoking (*P*=0.213) or alcohol (*P*=0.107) consumption was seen between the subjects with LULs and those without.

### Histologic and clinicopathologic findings

The samples of LULs consisted of 137 NDE and 15 dysplastic samples, whereas no dysplastic samples were detected in the normal Lugol staining samples (Table 1). Whereas the histologic finding in all samples of LULs-NDE was oesophagitis, 78% of the 103 normal Lugol staining epithelium samples were oesophagitis (*P*<0.0001). The histologic grade of dysplastic samples was low-grade in nine of 15 (60%) samples and high-grade in six (40%) samples.

The clinicopathologic findings of LULs-NDE and dysplasia are shown in Table 2. Most LULs-NDE and dysplasia also were located in the middle third of the thoracic oesophagus, as most invasive ESCCs were located in the same portion (Registration Committee for Esophageal Cancer). The characteristics of LULs-NDE were minute size (<5 mm in diameter), oval shape, and location in the middle third of the oesophagus.

### Mutation of the *p53* gene

*p53* mutation was detected in five of the 137 LULs-NDE samples, whereas no *p53* mutations were found in normal Lugol staining epithelium samples (Table 2). The mutations of the *p53* gene in LULs-NDE were one in exon 6, three in exon 7, and one in exon 8, and all were missense mutations (Table 3). A ‘hotspot’ mutation at codon 273 was found in one of the five LULs-NDE. A *p53* mutation was found in three of nine subjects (33%) with low-grade dysplasia and two of six subjects (33%) with high-grade dysplasia. The mutations of the *p53* gene in dysplastic lesions were three in exon 5 and two in exon 6, and four were missense mutations and one was a nonsense mutation resulting in insertion of a stop codon. A hotspot mutation at codon 175 was found in two of five dysplasia samples and these two samples were low-grade dysplasia.

**Figure 3:**
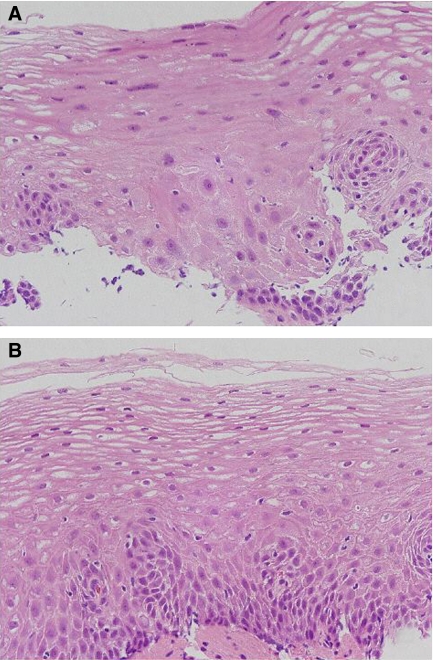
(**A**) Histologic findings of squamous atypia in a Lugol-unstained lesion with *p53* mutation. The region with squamous atypia was a small portion in contact with the basal cell layer. In the region, the nucleus was slightly enlarged, whereas pleomorphism and hyperchromasia were not seen. According to histological criteria of the Chinese group, the findings of slightly mononuclear enlargement having neither pleomorphism nor hyperchromasia were insufficient for diagnosis of dysplasia, and was decided as inflammation containing atypia. (**B**) Histologic findings of no squamous atypia in a Lugol-unstained lesion with *p53* mutation. Of the five Lugol-unstained lesions with non-dysplastic epithelium (LULs-NDE) containing *p53* mutation, squamous atypia was not found in four LULs-NDE.

In contrast, 22 (16%) of 137 LULs-NDE showed squamous atypia (Table 4). *p53* mutation was found in one (4.5%) of 22 LULs-NDE with squamous atypia (Figure 3A), and in four (3.5%) of 115 LULs-NDE without squamous atypia (Figure 3B and Table 4). Approximately 80% of the normal Lugol staining epithelium samples were oesophagitis, whereas no squamous atypia was found in normal epithelium samples.

Basal cell hyperplasia was present in 2% of LUL samples and 2% of normal Lugol staining epithelium samples alone (Table 1). Notably, *p53* mutations were not found in both LULs-NDE and normal Lugol staining epithelium samples with BCH (Table 4).

### Multiple LULs

A single or few LULs were detected in 98 (95%) subjects and multiple LULs were found in five (5%) subjects (Table 5). Although multiple LULs were found in only five (0.9%) of the 542 subjects, three of the five subjects with multiple LULs (60%) had dysplasia (*P*=0.003; Table 5).

No significant difference was seen in the occurrence of *p53* mutations between subjects with single or few LULs and multiple LULs (Table 5). Although the same mutation in exon 6 at (codon 218) was found in both dysplasia and LULs-NDE in case 2 with multiple LULs-NDE (Table 3), the two lesions were independent and were not contiguous. One lesion was located in the middle oesophagus, with dysplasia of 6-mm diameter area; the other was located in the lower oesophagus, with LUL-NDE of 4-mm diameter area.

## DISCUSSION

This study is aimed to evaluate whether LULs-NDE are related to the carcinogenesis of oesophageal squamous epithelium or not, and *p53* mutational status in LULs-NDE is analysed on the basis of molecular events in the progressive process of carcinoma. As *p53* mutation is a well-known sequence in dysplasia and carcinoma, this biomarker was determined to identify the precancerous lesions in the study. The unique observation in this prospective study is that missense mutations of the *p53* gene were found in LULs-NDE, although no *p53* mutations were found in paired normally Lugol-stained non-dysplastic epithelium in subjects with LULs-NDE. The results strongly suggest that some of the LULs-NDE can progress to dysplastic lesions through *p53* alterations and support the hypothesis that some of ‘Lugol-unstained non-dysplastic areas’ in Japanese individuals without reflux esophagitis play an important role in oesophageal carcinogenesis.

Mutations of the *p53* tumour suppressor gene are the most common genetic abnormalities in solid human cancers (Nigro *et al*, 1989; Hollstein *et al*, 1990; Lane, 1992; Vogelstein *et al*, 2000; Vousden and Lu, 2002; Oliver *et al*, 2004). Missense mutations are found in 78% of the 6177 somatic *p53* mutations in exons 5–8 (Hussain and Harris, 1999), suggesting a correlation between the degree of evolutionary diversity and the structural or functional importance of individual amino-acid residues (Greenblatt *et al*, 1994). The change of protein structure or function caused by the individual amino-acid residues in LULs-NDE might be early molecular events in carcinogenesis. In contrast, *p53* gene mutations have been proposed to be concentrated in six hotspots (Hainaut *et al*, 1997; Hussain and Harris, 1999; Vikhanskaya *et al*, 2005). Based on the updated *p53* Gene Mutation Database containing 5961 mutations, codons 175, 245, 248, 249, 273, and 282 have been identified as mutation hotspots in human cancers, and the incidence of the hotspot mutations is specific molecular alterations in solid human cancers (Hainaut *et al*, 1997). A hotspot can identify a relationship between the mutation, protein structure and function, and carcinogenesis (Hsu *et al*, 1991; Cho *et al*, 1994; Greenblatt *et al*, 1994; Tornaletti and Pfeifer, 1994). Furthermore, hotspot mutations in carcinomas represent protein alterations that provide a selective growth advantage to the cell, and missense mutations at six hotspots account for 25–30% of the mutations (Greenblatt *et al*, 1994; Hainaut *et al*, 1997; Hussain and Harris, 1999; Ito *et al*, 2000). Therefore, protein alterations that provide a selective growth advantage to the cell would have already occurred in cells of LULs-NDE before histologic transformation into dysplastic cells. Mutations at codon 175 and 273 have been shown to have transforming frequencies that are 22- and eight-fold, respectively, the basal level of wild-type p53 protein (Zambetti and Levine, 1993). From our results, the LUL-NDE or low-grade dysplasia containing mutations with high transforming activities, such as codon 175 and 273 mutations, might have growth advantages favouring progression to invasive ESCC with the acquisition of other genetic changes, and may acquire malignant potential before morphologically manifested cell proliferation at an early molecular level of carcinogenesis.

One group has proposed that BCH is an early indicator of oesophageal carcinogenesis (Yang, 1980; Yang and Qiu, 1987; Qiu and Yang, 1988; Wang *et al*, 1990). Wang *et al* (1996) reported that BCH can be found in 69% of biopsy samples in symptom-free patients and that *p53* mutations can be found in BCH and dysplastic samples, whereas no hotspot mutations are contained in these mutations (Shi *et al*, 1999). We also identified the histologic findings of BCH in LULs-NDE and the paired normal Lugol staining area according to histologic criteria used in the Chinese group (Dawsey *et al*, 1994), whereas prevalence of BCH was low in our Japanese subjects and no *p53* mutations were found. We do not believe that the role of BCH is related to oesophageal carcinogenesis in the Japanese population. In contrast, we did not suggest that the daily cigarette or alcohol consumption was directly related to the occurrence of LULs-NDE in this study despite high risk factors in patients with ESCC.

Using Lugol solution spraying methods, as the normal squamous epithelium contains glycogen that interacts with the iodine of Lugol's solution, normal epithelium of the oesophagus becomes uniformly greenish brown (Sugimach *et al*, 1991; Katagiri *et al*, 2004). Dysplastic and inflammatory epithelia of the oesophagus are not stained, as the region showing dysplasia and oesophagitis has a reduced or no glycogen content (Sugimach *et al*, 1991). Therefore, these minute lesions that were not identifiable by conventional endoscopic observation become visible when Lugol's solution is used. There is a high possibility that inflammation having a reduction in glycogen content is related to the initiation of oesophageal carcinogenesis because no squamous atypia and no *p53* mutations are found in normal Lugol staining areas with sufficient glycogen content. Squamous atypia would be transitional lesions from oesophagitis to dysplasia.

Although the prevalence of multiple LULs was low in oesophageal cancer-free subjects (0.9%), dysplasia occurred frequently in subjects with multiple LULs (60%). Muto *et al* (2002) reported that multiple LULs were found in 27% of head and neck cancer patients, and secondary ESCCs were found in 72% of such cancer patients with multiple LULs. They provided essential information about field cancerisation and malignant potential with respect to multiple LULs. The field cancerisation phenomena proposed that multiple squamous cell carcinomas occurred either simultaneously with the primary lesion (synchronous) or after a period of time (metachronous) in the oesophagus and the head and neck region. There is a possibility that widespread epithelial oncogenic alterations were found in patients with multiple LULs. In case 2, the same mutation at codon 218 was found in both LUL-NDE and dysplastic lesion, whereas *p53* mutation was not detected in background normal Lugol staining epithelium. The *p53* mutational status, in this case, reflects the phenomena of field cancerisation, which can be considered as high malignant potential.

The *p53* missense mutations containing a hotspot mutation were found in LULs-NDE in oesophageal cancer-free individuals without reflux oesophagitis. The finding suggests that LUL-NDE is an initial lesion for oesophageal carcinogenesis, and that the role of BCH is less clear for oesophageal carcinogenesis in Japanese individuals. The characteristic findings of high-risk population of oesophageal carcinoma were evaluated by genetic analyses, because it appeared that we emphasise the importance of both endoscopic detection of LUL-NDE and molecular diagnosis. We concluded that the understanding of aetiology in human oesophageal precursor at the molecular level could provide essential information about the identification of useful biomarkers for prevention studies.

## Figures and Tables

**Table 1:** Histologic findings of biopsy samples from 103 oesophageal cancer-free patients

	**Lugol-unstained lesions**	**Normal Lugol staining epithelium**	***P*-value**
Number of samples	152	103	
			
*Histologic findings*			
Dysplasia	15	—	
Oesophagitis	137	80	<0.0001
Normal epithelium	—	23	
			
*Basal cell hyperplasia*			
Present	3	2	0.986
Absent	149	101	

**Table 2:** Clinicopathologic characteristics and presence of *p53* mutation of LUL-NDE and dysplasia

	**LUL-NDE (*n*=137)**	**Dysplasia (*n*=15)**	***P*-value**
Mean size (mm)	4	9	0.032
Range (mm)	1–6	5–20	
			
*Shape of LUL*			
Oval	108	5	<0.0001
Irregular	29	10	
			
*Location*			
Upper third	19	1	0.441
Middle third	90	9	
Lower third	28	5	
			
*P53 mutation*			
Present	5	5	<0.0001
Absent	132	10	
			
*Hotspot mutation (10 samples with p53 mutation)*			
Present	1	2	0.490
Absent	4	3	

LUL-NDE=Lugol-unstained lesion with non-dysplastic epithelium; location=location of the oesophagus.

**Table 3:** Mutation of the *p53* gene in patients with Lugol-unstained lesions

**Case**	**Age/sex**	**Histology**	**Size (mm)**	**p53**	**Ex**	**Codon**	**BC**	**AAC**
1	52/M	itis	3	P	7	242	TGC → TCC	K → S
2	53/M	sev, dys.	6	P	6	218	GTG → GAG	V → E
		itis	4	P	6	218	GTG → GAG	V → E
		itis	3	A				
3	78/M	mod, dys.	0	P	6	192	CAG → TAG	^*^
4	71/M	mild, dys.	8	P	5	175	CGC → CAC	R → H
5	53/F	mild, dys.	6	P	5	175	CGC → GGC	R → G
6	63/M	sev, dys.	13	P	5	184	GAT → AAT	D → N
7	68/F	itis	3	P	7	241	TCC → TAC	S → Y
8	75/M	itis	4	P	8	273	CGT → TGT	R → C
		itis	2	A				
9	60/F	itis	4	P	7	239	AAC → GAC	N → D
		itis	5	A				
		itis	3	A				

itis=oesophagitis; dys, dysplasia; sev=severely; mod=moderately; P=presence of a *p53* mutation; A=absence of a *p53* mutation; EX=exon; BC=base change; AAC=amino-acid change; ^*^=stop codon.

**Table 4:** Relationship between presence of *p53* mutation and BCH or squamous atypia in 137 LULs-NDE

	**p53 mutation**	**p53 mutation**	
	**Present (*n*=5)**	**Absent (*n*=132)**	***P*-value**
*Squamous atypia*			
Present (*n*=22)	1	21	0.807
Absent (*n*=115)	4	111	
			
*BCH*			
Present (*n*=3)	0	3	0.733
Absent (*n*=134)	5	129	

LULs-NDE=Lugol-unstained lesions with non-dysplastic epithelium; BCH=basal cell hyperplasia.

**Table 5:** Presence of dysplasia and *p53* mutation between single or few and multiple Lugol-unstained lesions in 103 patients

	**Single or few LULs (*n*=98)**	**Multiple LULs (*n*=5)**	***P*-value**
*Dysplasia*			
Present	12 (12%)	3 (60%)	0.003
Absent	86 (88%)	2 (40%)	
			
*p53 mutation*			
Present	8 (8%)	1 (20%)	0.361
Absent	90 (92%)	4 (80%)	

LULs=Lugol-unstained lesions.

## References

[bib1] Bennett WP, Hollstein MC, Metcalf RA, Welsh JA, He A, Zhu SM, Kusters I, Resau JH, Trump BF, Lane DP (1992) p53 mutation and protein accumulation during multistage human esophageal carcinogenesis. Cancer Res 52: 6092–6097 1394236

[bib2] Cho Y, Gorina S, Jeffrey PD, Pavletich NP (1994) Crystal structure of a p53 tumor suppressor-DNA complex: understanding tumorigenic mutations. Science 265: 346–355 8023157 10.1126/science.8023157

[bib3] Dawsey SM, Fleischer DE, Wang GQ, Zhou B, Kidwell JA, Lu N, Lewin KJ, Roth MJ, Tio TL, Taylor PR (1998) Mucosal iodine staining improves endoscopic visualization of squamous dysplasia and squamous cell carcinoma of the esophagus in Linxian, China. Cancer 83: 220–231 9669803

[bib4] Dawsey SM, Lewin KJ, Liu FS, Wang GQ, Shen Q (1994) Esophageal morphology from Linxian, China. Squamous histologic findings in 754 patients. Cancer 73: 2027–2037 8156507 10.1002/1097-0142(19940415)73:8<2027::aid-cncr2820730803>3.0.co;2-3

[bib5] Gao H, Wang LD, Zhou Q, Hong JY, Huang TY, Yang CS (1994) p53 tumor suppressor gene mutation in early esophageal precancerous lesions and carcinoma among high-risk populations in Henan, China. Cancer Res 54: 4342–4346 8044781

[bib6] Goelz SE, Hamilton SR, Vogelstein B (1985) Purification of DNA from formaldehyde fixed and paraffin embedded human tissue. Biochem Biophys Res Commun 130: 118–126 2992457 10.1016/0006-291x(85)90390-0

[bib7] Greenblatt MS, Bennett WP, Hollstein M, Harris CC (1994) Mutations in the p53 tumor suppressor gene: clues to cancer etiology and molecular pathogenesis. Cancer Res 54: 4855–4878 8069852

[bib8] Hainaut P, Soussi T, Shomer B, Hollstein M, Greenblatt M, Hovig E, Harris CC, Montesano R (1997) Database p53 gene somatic mutation in human tumors and cell lines: updated complication and future prospects. Nucleic Acid Res 25: 151–157 9016527 10.1093/nar/25.1.151 PMC146396

[bib9] Hollstein MC, Metcalf RA, Welsh JA, Montesano R, Harris CC (1990) Frequent mutation of the p53 gene in human esophageal cancer. Proc Natl Acad Sci USA 87: 9958–9961 2263646 10.1073/pnas.87.24.9958 PMC55293

[bib10] Hsu IC, Metcalf RA, Sun T, Welsh JA, Wang NJ, Harris CC (1991) Mutational hotspot in the p53 gene in human hepatocellular carcinomas. Nature 350: 427–428 1849234 10.1038/350427a0

[bib11] Hussain SP, Harris CC (1999) p53 mutation spectrum and load: the generation of hypotheses linking the exposure of endogenous or exogenous carcinogens to human cancer. Mutat Res 428: 23–32 10517975 10.1016/s1383-5742(99)00028-9

[bib12] Ito T, Kaneko K, Makino R, Ito H, Konishi K, Kurahashi T, Kitahara T, Mitamura K (2000) Prognostic value of p53 mutations in patients with locally advanced esophageal carcinoma treated with definitive chemoradiotherapy. J Gastroenterol 36: 303–311 10.1007/s005350170095 11388392

[bib13] Jaskiewicz K, De Groot K (1994) p53 gene mutants expression, cellular proliferation and differentiation in esophageal carcinoma and non-cancerous epithelium. Anticancer Res 14: 137–140 8166440

[bib14] Katagiri A, Kaneko K, Konishi K, Ito H, Kushima M, Mitamura K (2004) Lugol staining pattern in background epithelium of patients with esophageal squamous cell carcinoma. Hepatogastroenterology 51: 713–717 15143899

[bib15] Kumagai Y, Makuuchi H, Mitomi T, Ohmori T (1993) A new classification system for early carcinomas of the esophagus. Dig Endosc 15: 5139–5150

[bib16] Lane DP (1992) p53 guardian of the genome. Nature (London) 358: 15–16 1614522 10.1038/358015a0

[bib17] Makino R, Kaneko K, Kurahashi T, Matsumura T, Mitamura K (2000) Detection of mutation of the p53 gene with high sensitivity by fluorescence-based PCR-SSCP analysis using low-pH buffer and an automated DNA sequencer in a large number of DNA samples. Mutat Res 452: 83–90 10894894 10.1016/s0027-5107(00)00056-7

[bib18] Mandard AM, Hainaut P, Hollstein M (2000) Genetic steps in the development of squamous cell carcinoma of the esophagus. Mutat Res 462: 335–342 10767643 10.1016/s1383-5742(00)00019-3

[bib19] Meyer V, Burtin P, Bour B, Blanchi A, Cales P, Oberti F, Person B, Croue A, Dohn S, Benoit R, Fabiani B, Boyer J (1997) Endoscopic detection of early esophageal cancer in a high-risk population: does Lugol staining improve videoendoscopy? Gastrointest Endosc 45: 480–484 9199904 10.1016/s0016-5107(97)70177-9

[bib20] Mori M, Adachi Y, Matsushima T, Matsuda H, Kuwano H, Sugimachi K (1993) Lugol staining pattern and histology of esophageal lesions. Am J Gastroenterol 88: 701–705 7683176

[bib21] Muto M, Nakane M, Hitomi Y, Yoshida S, Sasaki S, Yoshida S, Ebihara S, Esumi H (2002) Association between aldehyde dehydrogenase gene polymorphisms and the phenomenon of field cancerization in patients with head and neck cancer. Carcinogenesis 23: 1759–1765 12376487 10.1093/carcin/23.10.1759

[bib22] Nigro JM, Baker SJ, Preisinger AC, Jessup JM, Hostetter R, Cleary K, Bigner SH, Davidson N, Baylin S, Sevilee P, Glver T, Collins FS, Weston A, Modali R, Harris CC, Vogelstein B (1989) Mutations in the p53 gene occur in diverse human tumor types. Nature (London) 342: 705–708 2531845 10.1038/342705a0

[bib23] Oliver M, Hussain SP, Caron de Fromentel C, Hainaut P, Harris CC (2004) TP53 mutation spectra and load: a tool for generating hypotheses on the etiology of cancer. IARC Sci Publ 157: 247–270 15055300

[bib24] Parenti AR, Rugge M, Frizzera E, Ruol A, Noventa F, Ancona E, Ninfo V (1995) p53 overexpression in the multistep process of esophageal carcinogenesis. Am J Surg Pathol 19: 1418–1422 7503363 10.1097/00000478-199512000-00008

[bib25] Parkin DM, Laara E, Muir CS (1988) Estimates of the worldwide frequency of 16 major cancers in 1980s. Int J Cancer 41: 184–197 3338870 10.1002/ijc.2910410205

[bib26] Qiu SL, Yang GR (1988) Precursor lesions of esophageal cancer in high-risk populations in henan province, China. Cancer 62: 551–557 3390795 10.1002/1097-0142(19880801)62:3<551::aid-cncr2820620319>3.0.co;2-y

[bib27] Registration Committee for Esophageal Cancer (ed) Comprehensive registry of esophageal cancer in Japan. The Japan Society for Esophageal Diseases, http://plaza.umin.ac.jp/_~jsed/

[bib28] Shi ST, Yang GY, Wang LD, Xue Z, Feng B, Ding W, Xing EP, Yang CS (1999) Role of p53 gene mutations in human esophageal carcinogenesis: results from immunohistochemical and mutation analyses of carcinomas and nearby non-cancerous lesions. Carcinogenesis 20: 591–597 10223186 10.1093/carcin/20.4.591

[bib29] Sugimach K, Tsutsui S, Kitamura K, Morita M, Mori M, Kuwano H (1991) Lugol stain for intraoperative determination of the proximal surgical margin of the esophagus. J Surg Oncol 46: 226–229 1706814 10.1002/jso.2930460404

[bib30] Sugimachi K, Ohno S, Matsuda H, Mori M, Matsuoka H, Kuwano H (1989) Clinicopathologic study of early stage esophageal carcinoma. Surgery 105: 706–710 2727899

[bib31] Tornaletti S, Pfeifer GP (1994) Slow repair of pyrimidine dimers at p53 mutation hotspots in skin cancer. Science 263: 1436–1438 8128225 10.1126/science.8128225

[bib32] Vikhanskaya F, Siddique MM, Lee MK, Broggini M, Sabapathy K (2005) Evaluation of the combined effect of p53 codon 72 polymorphism and hotspot mutations in response to anticancer drugs. Clin Cancer Res 11: 4348–4356 15958617 10.1158/1078-0432.CCR-04-1547

[bib33] Vogelstein B, Lane D, Levine AJ (2000) Surface the p53 network. Nature 408: 307–310 11099028 10.1038/35042675

[bib34] Von Rensburg SJ (1981) Epidemiologic and ditary evidence for a specific nutritional predisposition to esophageal cancer. J Natl Cancer Inst 67: 243–251 6943364

[bib35] Vousden KH, Lu X (2002) Live or die: the cell's response to p53. Nat Rev Cancer 2: 594–604 12154352 10.1038/nrc864

[bib36] Wang LD, Hong JY, Qiu SL, Gao H, Yang CS (1993) Accumulation of p53 protein in human esophageal precancerous lesions: a possible early biomarker for carcinogenesis. Cancer Res 53: 1783–1787 8467496

[bib37] Wang LD, Lipkin M, Qiu SL, Yang GR, Yang CS, Newmark HL (1990) Labeling index and labeling distribution of cells in the esophageal epithelium in individuals at increased risk for esophageal cancer in Huixian, China. Cancer Res 50: 2651–2653 2328490

[bib38] Wang LD, Zhou Q, Hong JY, Qiu SL, Yang CS (1996) p53 protein accumulation and gene mutations in multifocal esophageal precancerous lesions from symptom free subjects in a high incidence area for esophageal carcinoma in Henan, China. Cancer 77: 1244–1249 8608498

[bib39] Yang CS (1980) Research on esophageal cancer in China: a review. Cancer Res 40: 2633–2644 6992989

[bib40] Yang GR, Qiu SL (1987) Endoscopic surveys in high-risk populations for esophageal cancer in China with special reference to precursors of esophageal cancer. Endoscopy 19: 91–95 3608927 10.1055/s-2007-1018250

[bib41] Yokoyama A, Ohmori T, Makuuchi H, Maruyama K, Okuyama K, Takahashi H, Yokoyama T, Yoshino K, Hayashida M, Ishii H (1995) Successful screening for early esophageal cancer in alcoholics using endoscopy and mucosal iodine staining. Cancer 76: 928–934 8625217 10.1002/1097-0142(19950915)76:6<928::aid-cncr2820760604>3.0.co;2-5

[bib42] Yoshinaka H, Shimazu H, Fukumoto T, Baba M (1991) Superficial esophageal carcinoma: a clinicopathological review of 59 cases. Am J Gastroenterol 86: 1413–1418 1656727

[bib43] Zambetti GP, Levine AJ (1993) A comparison of the biological activities of wild-type and mutant p53. FASEB J 7: 855–865 8344485 10.1096/fasebj.7.10.8344485

